# The Vulnerability of the Power Grid Structure: A System Analysis Based on Complex Network Theory

**DOI:** 10.3390/s21217097

**Published:** 2021-10-26

**Authors:** Banghua Xie, Xiaoge Tian, Liulin Kong, Weiming Chen

**Affiliations:** Faculty of Engineering, China University of Geosciences, Wuhan 430074, China; xiebanghua@cug.edu.cn (B.X.); xiaogetian@cug.edu.cn (X.T.); chenweiming@cug.edu.cn (W.C.)

**Keywords:** extreme weather, cascading failure, structural vulnerability, complex network, attack modes

## Abstract

The safety and reliability of the power grid are related to national power security, economic development and people’s daily life. The occurrence of extreme weather changes the external environment greatly. Including generators and transmission lines, many power grid units cannot resist such a huge attack and get damaged easily, which forces units to quit from the power grid running system for a while. Furthermore, if the number of influenced units is high enough, the whole power system will be destroyed by cascading failure caused by extreme weather. Aiming at dealing with the cascading failure emergencies, this paper is trying to improve the traditional power structural vulnerability model so that it can be used to discuss extreme weather and propose a theoretical topological model to help scholars measure the damage caused by extreme cases. Based on previous research in this field, this paper utilizes complex network knowledge to build the power grid topology model. Then, considering extreme cases and the three attack modes simulation process, this paper makes use of the characteristic parameters of the power grid topology model and designs an algorithm, according to the realistic situation of the propagation mechanism of cascading failure of the power grid model as well as extreme weather research. Finally, taking IEEE-30 and IEEE-118 node bus system as examples, which shows that the structural vulnerability method proposed in this paper can properly address the mechanism of unbalanced load of cascading failure of power grid units under extreme conditions and can provide theoretical reference for preventing and reducing the impact of extreme cases on power grid which improves the reliability of the power grid.

## 1. Introduction

Recently, global climate change brings new challenges to the power grid design [1]. Determining how best to address the impact brought by climate change is a problem. The literature [2] reviews methods used to model the extreme weather impact. In China, typhoons threaten the southern power system’s stability. Literature [3] summarizes and analyzes the wind accidents of the transmission line. As an emerging concept, [4] resilience is used to measure whether the power grid can be resilient to high-impact and low-probability events.

To be concrete, the state of power grid system and the reliability level of power grid are different under different situations [5], especially under extreme cases. To explore the way of quantifying impact of extreme weather, literature [6] proposes a quantitative reliability index to address the probability and result of power failures. Extreme weather can make the power grid units (including topological nodes and lines) much more unstable in a short time [7], which leads to the failure of individual or local units due to disturbance. As a result, the unit quits from the power grid system, changing the power flow, and then leads to cascade effect, which makes the more power units fail and out of control, leaving the power grid to collapse [8]. Taking power grid faults in central China as an example, in one-year, the number of faults of 500 kV transmission lines have reached 726 for extreme situations, and the faults of 220 kV transmission lines have reached 3668 [9]. Most of these cascading failures are usually caused by extreme cases. Once a key node or line failed and quit from the network, it would make other related nodes and lines get higher load pressure. The more key nodes or lines quit, the more major bottlenecks are created, which will contribute to the deterioration of electric parts like dielectric materials and operating systems [10]. Jufri, F.H. [11] concludes major power outages worldwide from 2011–2016 and the result showed as Figure 1, which shows that over 20 million people suffered from power outages during those five years and typhoon seems to be one of the most powerful extreme cases that causes severe power outages.

Hence, nowadays it is an urgent and important task to do research on analyzing the power grid structural vulnerability under risky circumstances. As one of the most crucial parts that affect the stability of the power grid, power grid vulnerability as a hot topic has widely concerned scholars at home and abroad. Some scholars try to propose methods for improving the resilience and stability of critical electrical power infrastructure to extreme weather [12], while others are trying to find indexes to address the topological vulnerability of the power grid. Particularly, complex network theory has been acknowledged in the wide domain for topological analysis of power systems [13]. Complex network, direct and alternating current power flow are common methods that can be used to model and establish the vulnerability model of the power grid. Literature [14] proposes a new vulnerability index which is weighted line betweenness to find critical lines in the power grid. Also, literature [15] proposes a new parameter named new-ability to evaluate the vulnerability of the power grid. Literature [16] does a survey to trace the evolution in the field where properties of different power grids are studied by using complex network analysis. Literature [17] reviews the latest and relevant papers that have studied robustness by using complex network theory, finding that few of papers focus on strategies to improve robustness. The research on vulnerability index is quite mature, such as load loss and transmission efficiency. The methods used for studying the power grid vulnerability can be classified as deterministic and uncertainty methods [18,19]. The deterministic methods are mainly based on operating state, while the uncertain methods refer to risk possibility assessment. Quantifying the impact of the weather is a difficult task due to its high stochasticity. Hence, a few literatures try to analyze the vulnerability under some attack modes.

Although the application of CN theory in the power system vulnerability assessment has drawn broad interest in recent years, challenges remain. Some literatures still focus on how to address the vulnerability or try to propose methods help improve the vulnerability of infrastructures of the power grid but do not consider structural vulnerability. Some try to analyze the structural vulnerability of the power grid by utilizing some simulation methods such as attack modes but do not consider the extreme cases. Some literatures consider the extreme cases and vulnerability of the power grid but they do not consider the simulation methods to test vulnerability such as attack modes. 

Thus, this paper considers the structural vulnerability of the power grid as well as extreme weather. Finally, this paper uses simulation methods to test and analyze the results. Compared with previously mentioned literatures, this paper not only focuses on vulnerability but also utilizes simulation methods of three special attack modes and extreme conditions. It provides a systematic view of how to analyze and test the vulnerability of the power grid under an extreme weather. Our research methods are as follows.

First, this paper establishes the whole power gird in a macro complex network perspective and improves the topological model by making it into a directed weighted topology model by classifying the node sources and weighting line reactance.

Also, characteristics and necessity of power grid vulnerability are obtained through several attack modes of power grid cascading failure, which is an effective way to quantify results. Based on this, this paper constructs the vulnerability model of power grid structure under extreme circumstances by using the theory of cascading failures.

Finally, extreme conditions are considered for analyzing structural vulnerability. The structural vulnerability model of the topology power grid is studied by utilizing the IEEE 118 bus node system. Results show that there is a significant relation between the structural vulnerability and extreme cases, proving our structural vulnerability model is useful and can be an example for the large-scale power grid. Figure 2 shows the structural organization of this paper:

## 2. Related Works

Vulnerability analysis mainly is studied by using below main methodologies [20]. First, define the structural, logical and functional relations among all units with the power grid. Second, try to quantify the performance of indicators. Third, studying system results under different accidental cases. Literatures aiming at vulnerability can be classified into two types [21]: 1. Analytical articles 2. Simulation articles. However, not all scholars can access a titanic amount of data from the real power grid. Hence, the simulation method is becoming more and more popular. Simulation methods can also be performed in two ways: Complex network and Direct current power flow. Ouyang, M. [22] compares three kinds of power grid models including topological model, betweenness based model and direct current power flow model in a vulnerability view to find that topological model and betweenness based model sometimes perform better when it comes to a certain marginal value. Methods in complex network can be divided into two major approaches: basic complex network models with few electrical elements and improved models with some features like betweenness added to the complex network [23]. This paper is proposing a power grid model considering network theory and typical research methods based on complex systems including cascade model, OPA model, branch process model, etc. [24]. Carreras, B. and Cao, Y. [25,26] analyze the power-law relationship between large-scale blackout and frequency in the American power system, proving it is a mathematical feature of self-organized criticality. Dwivedi A [27] proposes a max-flow-based complex network approach to analyze the vulnerability of power grid. Ren Z [28] counts relationship data between regional environment and line failure rate and uses the least square method to fit to obtain the grid unit failure rate. Besides, many literatures find that the power grid system obeys the small-world model and reveal that the influence of a few nodes and lines will cause great harm to the whole situation [29,30], which indicates the severe results that can be met by the power grid topological structure when it comes to some severe attack. Besides, there are many literatures concentrate on studying the algorithms that improve the complex network efficiency in the network theory [31,32,33]. Although these articles have studied systematically the complex network theory applications on the power grid, few of them pay much attention to analyzing the harsh outcomes met by the power grid. 

Most above literatures contribute more to the basic topological research method in establishing model, while other literatures focus on cascading failures which cause many blackouts accidents all around the world and find they can cause severe reduction in network stability as well as reliability [34,35]. Catastrophic outages caused by cascading power grid failures until now still lead to extremely serious effects. To prevent the power grid from cascading failures, analyzing, and measuring attacks that cause these problems is becoming an urgent topic. Liu, B. [36] utilizes complex network to analyze the vulnerability of key nodes of power grid and build a cascading failure model based on ac power flow model and network topology model, proving cascading failure can cause severe reduce in network stability and reliability. Li, K. and Liu, K. [37] first investigate the robustnesss of the Chinese power grid under different attack and defense effects and quantify two approaches to avoid this including improving load capacity and protecting critical edges. Wang, J.W. and Rong, L.L. [38] propose two attack strategies and quantified the results that indicate characteristics of a breakdown edge have an important impact on the effects of intentional attacks. Ding, M. [39] analyzes the influence of the overall structure of the power grid on the cascading failures of the power grid from the topology structure of the power grid, and points out that the higher number and degree of nodes will improve the connectivity of the power grid, but at the same time, the severity of the failures will be greater when the power grid is deliberately attacked. Power grid faults will affect the vulnerability of the power system, so it is necessary in order to analyze and evaluate its uncertainty and fault vulnerability, and consider the attacks suffered by the power grid [40,41]. Similar articles all contribute to propose new methods to protect the power grid network from cascading failures. However, few of them consider the possible concrete background of these attack conditions which makes the simulation or analysis lack of some credibility. 

In the view of the recoverability of the power grid system, some researchers focus on the measures to improve the flexibility of the power grid, including the preparation strategies before extreme situations and a series of operation strategies that can be adopted in some disasters [42,43,44,45,46,47]. Some researchers seek to propose various graded response mechanisms and recovery decision-making mechanisms after disasters [48,49,50,51]. While others focus on the research of power grid transmission framework topology such as power grid topology reconfiguration and dynamic topological analysis [52,53].

Hence, despite the fact that the above research method considers the failure rate of power grid units in extreme cases, it does not take into account the different action characteristics and effects of different extreme cases on the power grid. Using the constant failure rate model cannot reflect the randomness and characteristics of weather and cannot be well applied to unusual weather conditions and weather superposition

## 3. Background Knowledge

Wattes and Strogatz first introduce the small-world model, namely WS small-world model [54,55], and reveal that the small-world model exists in many common networks. Research shows that the power grid system also generally follows the small-world network model [56,57]. Their model is shown in Figure 3, where *p* refers to the possibility of randomness.

The above small-world network mainly introduces a small number of remote connections to the network through the process of edge reconnection, which makes the small-world network have the small characteristic path length of a random network. Therefore, once a small fault occurs in the power system, it may lead to a wide range of cascading failure, which may lead to the collapse of the power grid.

The large-scale interconnection of power grid makes it complicated to describe the dynamic situation of the power system. In addition, if local units are unable to exit the power grid due to low functional vulnerability, it is more likely to lead to continuous chain failure, resulting in large-scale power outages and heavy losses. To study the vulnerability of the power grid from a macro perspective, the first goal is to transform the case power grid structure into a topological model based on graph theory.

Then, when modeling according to the structure and characteristics of different types of power grid units, the power grid units are divided into two categories, which are represented by nodes and edges, respectively, which form a topological model without direction and weight. There are *k* edges numbered from 1 to *k*. Each edge can be labeled with *i,j*, which means the start node is node *i* and the end node is node *j*. We define an undirected unweighted graph called V=A,B like Figure 4, in which *A* and *B* are respectively defined as column matrix and n-order symmetric correlation matrices to represent, respectively, all nodes and the connection relationship between nodes. The element in the *i*th row and the *j*th column in the matrices *B* is the connectivity coefficient $b_{ij}$, while $a_{i^{\prime }}$ represents the $i^{\prime }$th node in n node. Therefore, the number of network edges *k* is equal to the number of elements when $b_{ij}=1\;\left(1\leq i,j\leq n\right)$. Also, if there is a node then $a_{i^{\prime }}=1$ else the $a_{i^{\prime }}=0\;\left(1\leq i^{\prime }\leq n\right)$. Or if there is a connection between *i* and *j* $b_{ij}=1$ else $b_{ij}=0$.

(1) $$A=\left(a_{1},a_{2}\cdots a_{n}\right),$$

(2) $$B=\left(\begin{array}{cccc} b_{11} & b_{12} & \cdots & b_{1n}\\ b_{21} & b_{22} & \cdots & b_{2n}\\ \vdots & \vdots & \ddots & \vdots \\ b_{n1} & b_{n2} & \cdots & b_{nn} \end{array}\right),$$

Energy flow can be in both directions along the edges of the network, and the weight of each edge is 1. When there is a parallel connection, because the number of multipath edges is the same and the multipath weight is the same, the minimum path cannot be determined, which has a great influence on the analysis of network parameters. So, in this paper, the undirected and unweighted model is changed into a directed and weighted one.

Energy can only be transferred from the node with higher electrical potential to the node with lower electrical potential, transforming the undirected topological model into the directed topological model. In this paper, grid nodes consist of three types as in Figure 5, which can quite match the reality. When the output power of nodes is greater than the load power, the nodes are the source nodes. When the output power of a node is lower than the load power, the node is a sink node. Additional nodes are contact nodes. 

As shown in Figure 5, if node *v* belongs to the source node, then the path only leaves from this node. This is because the source node is the starting node of the shortest path, and the number of shortest paths leaving the node is less than the number of shortest paths leaving the node. On the contrary, if node *v* belongs to a sink node, the number of shortest paths entering the node is greater than the number of shortest paths leaving the node. In this case, this paper introduces a virtual node to transform node *v* into a link node.

The reactance value $x_{ij0}$ is taken as the edge weight $w_{ij}$, and the reactance is similar to the effect of resistance on DC current. When the reactance value of the line is larger, the transmitted power is smaller, which makes the unweighted topology model be converted into the weighted topology model. Redefine an *N*-order weight matrix W to represent the connection relationship between nodes under weighting. The element in row *i* and column *j* of matrix *W* is $w_{ij}$:

(3) $$W=\left(\begin{array}{cccc} w_{11} & w_{12} & \cdots & w_{1n}\\ w_{21} & w_{22} & \cdots & w_{2n}\\ \vdots & \vdots & \ddots & \vdots \\ w_{n1} & w_{n2} & \cdots & w_{nn} \end{array}\right),$$

(4) $$w_{ij}=\left\{\begin{array}{c} 0,\;\;i=j\;\\ x_{ij},\;i\neq j\;and\;there\;is\;a\;\;connecting\;edge\;between\;i\;and\;j\\ \infty ,i\neq j\;and\;here\;is\;no\;\;connecting\;edge\;between\;i\;and\;j \end{array}\right.,$$

Distance $DIS_{ij}$ between node *i* and node *j* is the sum of the edge reactance included in the shortest path. Distance $DIS_{ik}$ between node *i* and *k* is obtained as Figure 6.

The average path length *AL* of the network is defined as the average distance between any two nodes.

(5) $$DIS_{ik}=w_{ij}+w_{jk},$$

(6) $$\frac{1}{2}n\left(n-1\right)AL=\underset{i\neq j}{\sum }DIS_{ij},$$

Two types of betweenness are divided into node betweenness $BT_{v}$ and edge betweenness $BT_{e}$. Edge betweenness $BT_{e}$ is the proportion of weighted shortest paths passing through an edge in the network to all weighted shortest paths in the network. Node betweenness $BT_{v}$ is the proportion of weighted shortest paths passing through a node in the network to all weighted shortest paths in the network, where $\delta_{ij}$ refers to the number of shortest paths between node *i* and node *j*. $\delta_{{ij}_{v}}$ and $\delta_{{ij}_{e}}$ are the number of shortest paths between node *i* and node *j* containing node *v* or edge *e,* respectively.

(7) $$BT_{v}=\frac{\sum_{i\neq v\neq j}\delta_{ij}{}_{v}}{\sum_{i\neq j}\delta_{ij}}$$

(8) $$BT_{e}=\frac{\sum_{i\neq j}\delta_{ij}{}_{e}}{\sum_{i\neq j}\delta_{ij}}$$

The betweenness $BT_{G}$ of source node *v* is as following, and *n_L_* is the number of sink nodes.

(9) $$BT_{G}=BT_{v}+\frac{1}{2}n_{L}$$

The betweenness $BT_{L}$ of sink node *v* is as following and *n_G_* is the number of source nodes.

(10) $$BT_{L}=BT_{v}+\frac{1}{2}n_{G}$$

In the reactance weighted network, the node degree *k_i_* remains unchanged, which is defined as the number of edges connected to this node. The average degree *AVED* of a network is the average degree of all node degree *k_i_*.

(11) $$AVED=\frac{1}{n}\sum k_{i}$$

The flow chart of power grid topology model is presented in Figure 7. It shows the process of power grid topology characteristic parameters and model modeling analysis as follows:

According to the relationship between energy inflow and outflow, the grid nodes are divided into source nodes, sink nodes and contact nodes, and the line reactance value is put in place to transform the model into a directed weighted model. By using the Floyd algorithm considering the previously mentioned parameters, the undirected and unweighted topological graph will become a directed and weighted topological graph. There will be three steps for this flow chart:

Step 1: Input data from IEEE-30 and IEEE-118.

Step 2: Traverse all lines and nodes in the graph and calculate the weight of each line of each node.

Step 3: Using Floyd algorithm to traverse all lines among nodes as the basic method, calculate the betweenness of three nodes: sources nodes, link nodes and sink nodes. After getting betweenness of all nodes, return and stop the algorithm.

## 4. Vulnerability of Power Grid Structure Based on Cascading Failures

**Figure 8:**
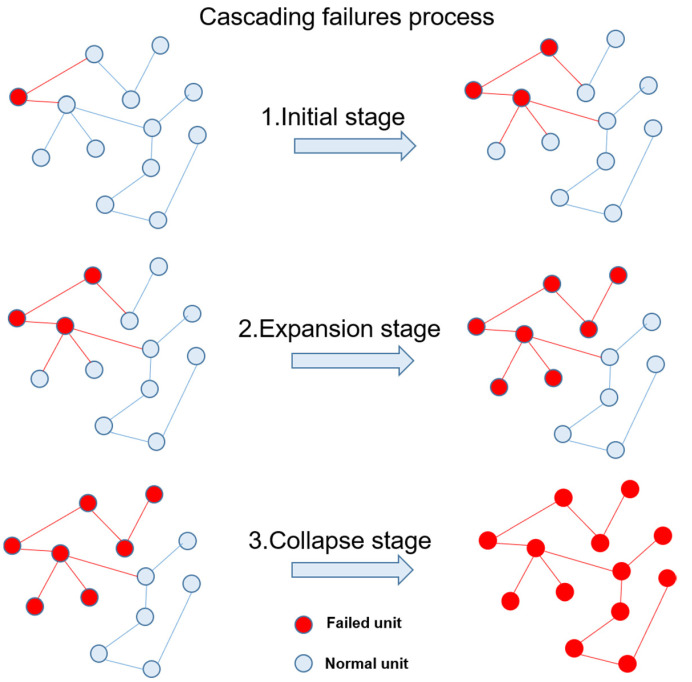
Cascading failure process.

Cascade failures refer to the fact that some units of the power grid exit the power grid due to slight disturbance, and the cascade effect causes the unbalanced load of other units in the neighborhood to cause faults, which eventually leads to the occurrence of large-scale power outages and makes the power grid collapse. The cascading failure process can be divided into three stages: initial stage, expansion stage, and collapse stage [58] in Figure 8:

1.  Initial stage: due to external disturbance in extreme cases, individual disturbed units fail due to limited capacity. In the initial stage of the accident, the whole power grid is less affected at this stage. If the accident is found in time and the corresponding preventive measures are taken, the further deterioration of the accident can be controlled in time;
2.  Expansion stage: in the initial stage, the fault continues to spread and expand, which leads to the change of unit load related to the unit logic of the initial fault under the action of system structural vulnerability, so that it is easy for the fault to exit the operation system. The expansion stage of accident expansion is formed by concluding which time the fault range of the power grid is expanded. However, it is still a partially controllable stage.
3.  Collapse stage: when the faults continue to spread and expand in the expansion stage, the loads caused by many local unit faults will accumulate further, which makes the overall load distribution and initial distribution of the system change rapidly. Due to the influence of the power grid, larger-scale faults will eventually lead to the cracking of the power grid system and even the whole power grid.

### 4.1. Evaluation Index of Power Grid Structural Vulnerability

The structural vulnerability of the power grid system is defined as the difficulty degree that individual units or subsystems in power grid system are disturbed and exit from operation, which makes the units or subsystems in the neighborhood exit one after another by a chain effect. Finally, it leads to the collapse and paralysis of the power grid system. In order to quantify the change degree of power grid transmission capacity before and after-failure due to vulnerability of power grid units, this paper defines the index of the power grid structural vulnerability as load loss percentage and network transmission efficiency, which are described as follows:

#### 4.1.1. Percentage of Load Loss

When the grid nodes are overloaded or run out of the grid due to the disturbance of the external environment, the load transmitted by the whole grid will change accordingly. $L_{j}$ and $L_{k}$ represent the load of *jth* and *kth* nodes. The percentage of load loss $\eta_{L}$ is defined as the ratio of the load of all normal operation sink nodes $L_{j}$ in the current power grid to the load of all nodes $L_{k}$ in the power grid in the initial state, which is expressed in a percentage system. *G*_1_ is the set of all failed transmission nodes. *G*_0_ is the set of all normal working nodes.

(12) $$\eta_{L}=\frac{\sum_{j\in G_{1}}L_{j}}{\sum_{k\in G_{0}}L_{k}}\times 100\%$$

#### 4.1.2. Network Transmission Efficiency

For the undirected powerless network topology model, the network transmission efficiency *E* is defined as the average of the reciprocal distance $DIS_{ij}$ between the network nodes of all node pairs in the power grid, i.e.,:

(13) $$E=\frac{1}{\frac{1}{2}n\left(n-1\right)}\underset{i\neq j}{\sum }\frac{1}{DIS_{ij}},$$

In this paper, the line reactance value is used as the weight of the edge, and the shortest transmission path of the node is the current transmission path. Using the line reactance value $w_{ij}$ instead of the network node distance $d_{ij}$ can better reflect the actual operation state of the power grid, so the network transmission efficiency *E* is overloaded as follows:

(14) $$E=\frac{1}{\frac{1}{2}n\left(n-1\right)}\underset{i\neq j}{\sum }\frac{1}{w_{ij}},$$

Assuming there is no direct connection between node pair *i* and *j*, it is known from the previous chapter that $w_{ij}\to \infty$ at this time and then $1/w_{ij}\to 0$.

The network transmission efficiency decline percentage $\eta_{E}$ is defined as the ratio between the current network transmission efficiency *E* and the initial network transmission efficiency $E_{0}$, which is expressed in percentage, namely:

(15) $$\eta_{E}=\frac{E}{E_{0}}\times 100\%$$

### 4.2. Vulnerability of Power Grid Structure

Due to the restriction of economic factors, the load capacity of the power grid unit is limited. The load on the line is the energy flowing through the line. Energy flowing through nodes can be split into inflow energy and outflow energy. Inflow energy is divided into generator input energy $L_{G}$ and input energy $L_{in}$, and outflow energy is node load energy $L_{l}$ and output energy $L_{out}$, as shown in Figure 9. The load of the node is all the energy flowing into or out of the node, i.e., The inflow load will always be equal to the outflow load according to basic Kirchhoff’s law of electrical circuits:

(16) $$L_{i}=L_{G}+L_{in}=L_{L}+L_{out}$$

In the grid cascading failure model, the load of each unit under the initial grid topology is taken as the reference load, and the rated standard load capacity $S_{Rated}{}_{i}$ and the limited load capacity $S_{Limit}{}_{i}$ are defined as follows:

(17) $$S_{Rated}{}_{i}=a_{0}\cdot L_{i0}$$

(18) $$S_{Limit}{}_{i}=a_{1}\cdot L_{i0}$$

$L_{i0}$ is the load of each unit under the initial power grid structure. $a_{0}$ is the rated load capacity coefficient. $a_{1}$ is the ultimate load capacity coefficient, which is taken as 1.1 and 1.2, respectively, in this paper.

The variation of external environmental factors and the fault of adjacent lines will make the load level of the line change [59]. Load specified capacity and load limit capacity are used to measure the impact of load on vulnerability, which cannot reflect the vulnerability level of power grid lines and systems in the short term in the future. Therefore, this paper improves the calculation method of unit failure rate of the line. Considering the functional relationship between the unit failure rate of the line and the load level, this paper divides the influence of the load on the failure rate into three piecewise functions and puts forward an improved cascading failure model of the power grid in extreme cases, which is described as follows [59]:

(19) $$\lambda_{i}\left(t\right)=\left\{\begin{array}{cc} \lambda_{i}{}_{0},\; & \;\;\;\;\;\;\;\;0<L_{i}\leq S_{Rated}{}_{i}\\ \frac{k}{{\left(S_{Limit}{}_{i}-L_{i}\right)}^{n}}+c, & \;\;\;\;\;\;\;\;S_{Rated}{}_{i}<L_{i}<S_{Limit}{}_{i}\;\\ \infty , & \;\;\;\;\;\;\;\;L_{i}\geq S_{Limit}{}_{i} \end{array}\right.$$

*Li* is the load value of line unit *I* under the current operating condition. $S_{Ratedi}$ is rated load capacity. $S_{Limiti}$ is the limit load capacity. *k*, *c,* and *n* are shape coefficient, position parameter and change coefficient, respectively. According to reference [59], the values of these three factors are 0.1$\lambda_{i0}$, 0, and 1, respectively.

When the load $L_{i}$ of power grid unit is less than the rated load capacity $S_{Ratedi}$, the failure rate of power grid lines is little affected by the load, which is the same as the original failure rate $\lambda_{i0}$ in the current state. When the power grid line unit exceeds the rated load capacity $S_{Ratedi}$ but does not exceed the limit load capacity $S_{Limiti}$, the line unit is in an overload state. When the adjacent lines quit operation, the system load will be redistributed, which will increase the transmission burden of other line units and increase the failure rate of line units. When the line load is closer to the limit load capacity $S_{Limiti}$, it is easier to receive external disturbances and cause faults. When the load $L_{i}$ of power grid unit exceeds the limit load capacity $S_{Limiti}$, the protection device cuts off the line.

When a grid unit is removed, the load of the unit will be distributed to other adjacent units according to the following equations. Based on the Kirchhoff’s laws using the node voltage method, the above DC resistive networks is established, in which the $w_{ij}$ refers to the component of the matrix mentioned before. The power flow of DC resistive networks can be obtained as follows:


(20) $$\left(\begin{array}{c} L_{1}\\ \vdots \\ L_{k}\\ \vdots \\ L_{n} \end{array}\right)=\left[\begin{array}{ccccc} w_{11} & \cdots & w_{1k} & \cdots & w_{1n}\\ \vdots & \ddots & \vdots & \ddots & \vdots \\ w_{k1} & \cdots & w_{kk} & \cdots & w_{kn}\\ \vdots & \ddots & \vdots & \ddots & \vdots \\ w_{n1} & \cdots & w_{nk} & \cdots & w_{nn} \end{array}\right]\left(\begin{array}{c} L_{{out}_{1}}\\ \vdots \\ L_{{out}_{k}}\\ \vdots \\ L_{{out}_{n}} \end{array}\right)$$

(21) $$L_{k}=L_{{out}_{k}}\sum_{m=1}^{n}W_{km}L_{out_{m}}\;$$

### 4.3. Extreme Weather Background

In this paper, the research of extreme weather is more about gale and icing weather which is common all over the world [60]. There are many literatures that focus on how to analyze extreme weather and its effects. Literature [61] proposes a circular geometrical model to address the wind and ice effects for their attributes. As a common phenomenon, ice will come with gusts, tornadoes, hurricanes, etc. Usually, icy weather and windy weather weaken the power grid in different ways. The main factor of icy weather that influences the power grid is ice coating the power wires, and that of windy weather is strong wind that can do damage to all wires and towers. Ice coating will result in the cross section of wires increasing and will affect the ice load. A heavy ice or snow event will collapse the high voltage power tower. Hence, in this paper, extreme cases will be set as wind and ice conditions and we build the extreme weather model:

(22) $$EW\left(wire_{x_{i}},wire_{y}{}_{i}\right)=Ae^{\left(-\frac{1}{2}\left({\left(\frac{wire_{x_{i}}-EW_{x}}{\partial r}\right)}^{2}+{\left(\frac{wire_{y_{i}}-EW_{y}}{\partial r}\right)}^{2}\right)\right)}$$

In the above (22), A denotes the extent of the extreme weather and $\left(wire_{x_{i}},wire_{y_{i}}\right)$ and $\left(EW_{x},EW_{y}\right),$ separately, represent the position of line/wire and extreme weather middle location. r is the radius of the extreme affecting range and ∂ is a coefficient. Since the extreme weather have already been classified two major types in this paper that will be studied, their power load model will also depend on both the extreme weather as well as their models. According to the literatures discussed before, here models can be built as follows:

(23) $$L_{I}=\int_{0}^{t}A_{I}e^{\left(-\frac{1}{2}\left({\left(\frac{wire_{x_{i}}-EW_{x}\left(t\right)}{\partial r}\right)}^{2}+{\left(\frac{wire_{y_{i}}-EW_{y}\left(t\right)}{\partial r}\right)}^{2}\right)\right)}dt$$

(24) $$L_{W}=W\left(t\right)\left(A_{W1}e^{\left(-\frac{1}{2}\left({\left(\frac{wire_{x_{i}}-EW_{x}\left(t\right)}{\partial r}\right)}^{2}+{\left(\frac{wire_{y_{i}}-EW_{y}\left(t\right)}{\partial r}\right)}^{2}\right)\right)}-A_{W2}e^{\left(-\frac{1}{2}\left({\left(\frac{wire_{x_{i}}-EW_{x}\left(t\right)}{\partial r_{1}}\right)}^{2}+{\left(\frac{wire_{y_{i}}-EW_{y}\left(t\right)}{\partial r_{1}}\right)}^{2}\right)\right)}\right),$$

The above Equations, $L_{I},$ and $L_{W}$ are load under icing and wind weather. (23) is in the form of integral for ice coating is constantly increasing. And in the next equation, $W\left(t\right)$ indicates the effects brought by wind angle for that only when the wind direction and the wires direction is at right angles the wires will suffer the whole effects. Otherwise, the wind can be treated as vector and be decomposed into two vectors, which includes a parallel one and a vector just at the right angles with the wire direction. This paper sets $\lambda_{I}\left(t\right)=g_{1}\left(L_{I}\left(t\right)\right)$ $\lambda_{W}\left(t\right)=g_{2}\left(L_{W}\left(t\right)\right)$, where $\lambda_{I}$ means the failure rate of ice load and $\lambda_{w}$ means the failure rate of wind load. $g_{1}$ and $g_{2}$ are used to express the two different failure rate function. Here comes a question: what about the regions that both suffer from ice and wind. $a_{I}$ and $a_{w}$ are the coefficients of the two weathers. Literature [61] suggest a coefficient that allows the two failure rates can add up to a new failure rate if there are two extreme conditions:

(25) $$\lambda =a_{I}\lambda_{I}\left(t\right)+a_{w}\lambda_{W}\left(t\right)$$

In extreme cases, when any unit of the power grid quits operation due to its own factors or disturbed faults, the load of the power grid unit is redistributed. When the load of adjacent units or small-scale subsystems exceeds the limit load, the fault occurs and is removed, which propagates repeatedly, resulting in cascading failure of the power grid.

### 4.4. Vulnerability Analysis Process of Power Grid Structure

In extreme cases, when any unit of the power grid quits operation due to its own factors or disturbed faults, the load of the power grid unit is redistributed. When the load of adjacent units or small-scale subsystems exceeds the limit load, the fault occurs and is removed, which propagates repeatedly, resulting in cascading failure of the power grid.

In order to discuss the vulnerability of small-world characteristics of power grid to power grid structure, this paper designs different attack strategies to attack power grid in extreme cases, so as to determine the impact of cascading failures on power grid structure vulnerability and find out the weak units or subsystems that have the greatest impact on the power grid.

This paper improves the attack mode of algorithm and divides it into the following three types for simulation:

(1) Line random attack: randomly attack a normal operation line according to the weight of line reliability and time is used to weigh the vulnerability of power grid function. This paper set the reliability as a time-based integration where $\lambda_{i}$ is unit failure rate:
  (26) $$F_{r}\left(t\right)=e^{\int_{0}^{t}\lambda_{i}dt}$$
(2) Line betweenness attack: attack the line with the largest specified betweenness in turn.
(3) linear function vulnerability attack: attack the line with the highest specified vulnerability in turn. The vulnerability is calculated by using load of loss. The vulnerability is calculated by using betweenness times weigh the vulnerability of power grid:

(27) $$F_{v}\left(t\right)=BT_{e}(1-e^{\int_{0}^{t}\lambda_{i}dt}),$$

**Figure 10:**
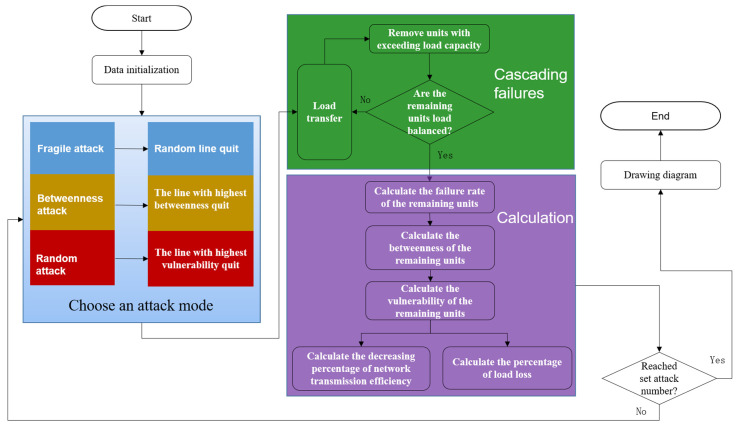
Flow chart of vulnerability analysis of power grid structure.

The process of vulnerability analysis of power grid structure in this paper. All the following steps of the analysis algorithms are established by using anaconda python 3 IDE. Steps are as follows and are shown in the Figure 10:

Step 1: Data initialization.

Input data from IEEE-30 and IEEE-118. At the same time, the extreme weather condition will also be included. Finally, set the number of the total loop.

Step 2: Choose an attack mode.

Enter the loop, and select the attack objects (the line with the highest vulnerability, the line with the highest intermediate number and the line with random attack) and exit the loop after attacks of settled number.

Step 3: Cascading failures.

Transfer the load of the line according to (21). According to the power dispatch after the load transferring, remove the nodes and related lines if the redistributed load exceeds the capacity of this node or line. From the first node and related lines to traverse to the last node and related lines to check whether the load of nodes and relative lines exceeds the capacity of nodes and lines. Set these nodes and lines as broken and remove them.

Step 4: Calculation.

After removing broken nodes and lines, it is necessary to recalculate the between-ness of remained nodes so that vulnerability parameters can be obtained later. Calculate the failure rate of load exceeding rated capacity but not exceeding limited capacity according to (19). Calculate the percentage of load loss and the variation ratio of network transmission efficiency according to (12)–(15).

In this paper, the index of structural vulnerability of the power grid with cascading failure is analyzed and reviewed. The percentage of load loss and network transmission efficiency are selected as indicators of structural vulnerability. The mechanism of cascading failure of the power grid under unbalanced load is explored. The connection between the unit load and power grid vulnerability in extreme cases is combined and the load distribution scheme under unbalanced load vulnerability is constructed. Simulation methods of deliberate attack and random attack are designed. In the fourth chapter, an example will be utilized to verify the rationality of the model constructed in this chapter and carry out risk assessment.

## 5. Example Analysis

In this paper, the IEEE-30 bus system and IEEE-118 bus system are taken as examples to analyze. The structural vulnerability of the power grid is calculated. Vulnerability of unbalanced power grid load in extreme cases is analyzed. Compared with IEEE-30, IEEE-118 represents a portion of the American Electric Power System (in the Midwestern US) as of December, 1962. It was entered in IEEE Common Data Format and PECO PSAP Format by Rich Christie at the University of Washington in 1993. The IEEE 30 Bus Test Case represents a portion of the American Electric Power System (in the Midwestern US) as of December, 1961. A hardcopy data was provided by Iraj Dabbagchi of AEP and entered in IEEE Common Data Format by Rich Christie at the University of Washington in August 1993. In this paper, the data of IEEE-30 and IEEE-118 is from “Ali R. Al-Roomi (2015). Power Flow Test Systems Repository (https://al-roomi.org/power-flow (accessed on 11 August 2021)). Halifax, Nova Scotia, Canada: Dalhousie University, Electrical and Computer Engineering.”

### 5.1. Topology Modeling and Analysis of Power Grid

#### 5.1.1. IEEE 30 Topology Model

Figure 11 is a wiring diagram of IEEE-30 node system, which consists of 30 nodes and 41 lines. According to the node properties mentioned above, the nodes can be divided into 5 source nodes, 19 sink nodes, and 6 contact nodes. Moreover, it is noted that although node 5 is both an engine node and a load node, the load power of node 5 should be greater than its generator input power in Table 1, so node 5 is a sink node. The specific type of each node is shown in Table 2. Label nodes and lines, respectively, and determine coordinate positions, and distinguish them with different colors according to node types.

According to the first chapter, the topology model of the power grid is established, and the power grid is transformed into a directed weighted topology model. Figure 11 is a schematic diagram of ice and wind effects range, in which a radius is established with a unit length of their effects.

#### 5.1.2. IEEE 118 Topology Model

**Figure 12:**
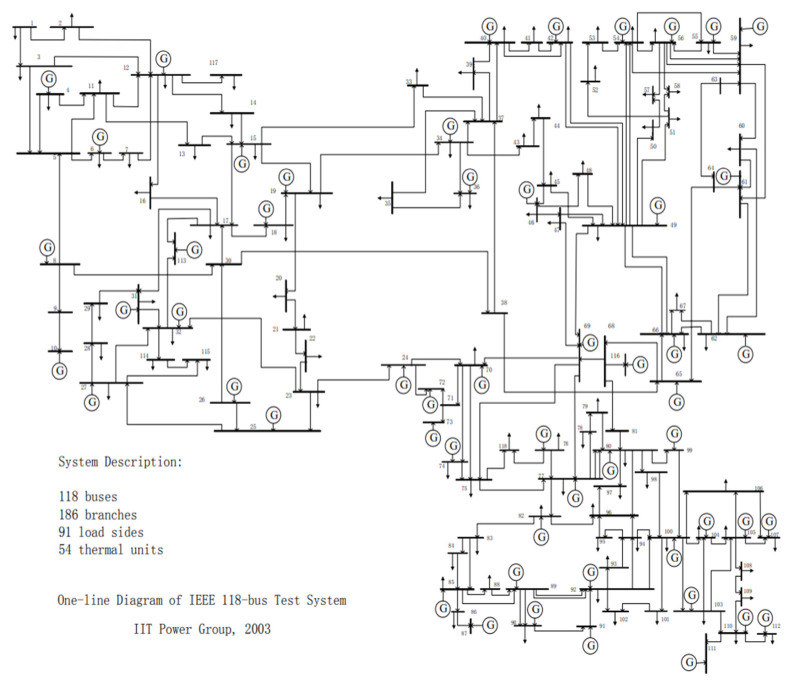
Standard IEEE118-node system.

Figure 12 is a wiring diagram of IEEE-118 node system. Label nodes and lines and determine positions. Finally, distinguish them with different colors according to node types and get pictures from 12 to 14.

**Figure 13:**
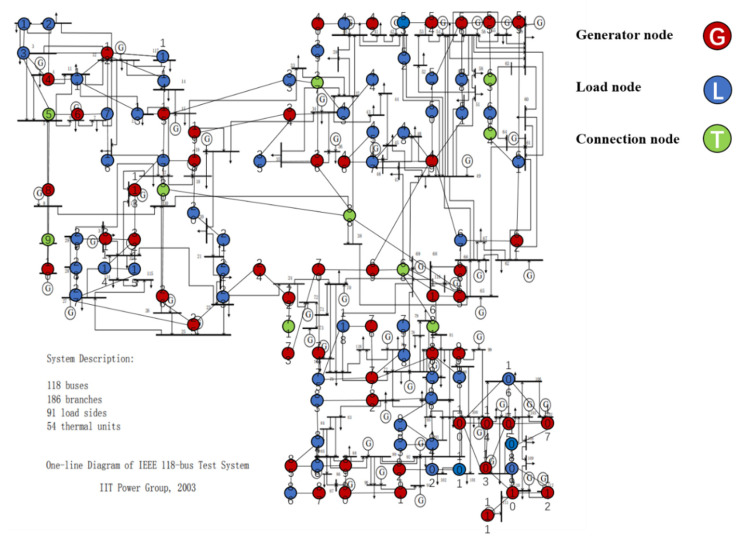
IEEE118-node system.

**Figure 14:**
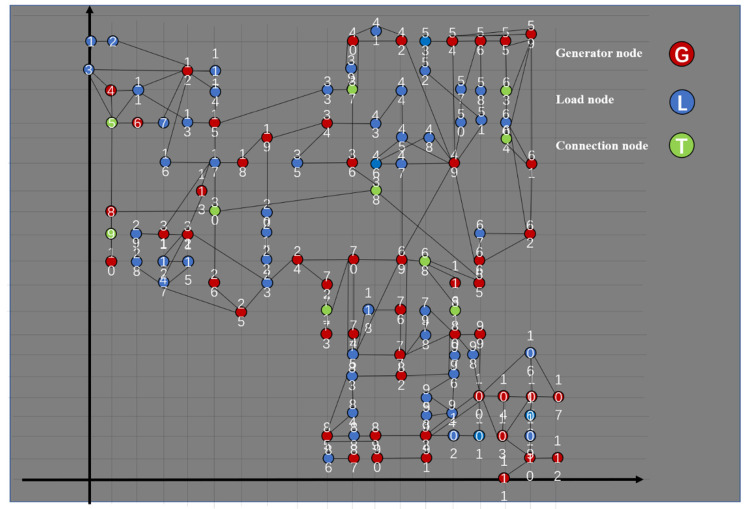
IEEE-118 bus system topology model diagram.

Figure 13 and Figure 14 are the topological diagrams and abstract topological with coordinates, which consists of 118 nodes and 186 lines. According to the node properties mentioned above, the nodes can be divided into 55 source nodes, 93 sink nodes, and 10 contact nodes. Moreover, there are some generators that do not generate power and they are sink nodes. The specific type of each node is shown in Table 3.

**Table 2:** IEEE-118 system node type.

Node Type	Node Number	Node ID
Source node	15	10, 12, 25, 26, 31, 49, 61, 65, 66, 69, 80, 87, 89, 100, 103
Sink node	93	1, 2, 3, 4, 6, 7, 8, 11, 13, 14, 15, 16, 17, 18, 19, 20, 21, 22, 23, 24, 27, 28, 29, 32, 33, 34, 35, 36, 39, 40, 41, 42, 43, 44, 45,46, 47, 48, 50, 51, 52, 53, 54, 55, 56, 57, 58, 59, 60, 62, 67, 70, 72, 73, 74, 75, 76, 77, 78, 79, 82, 83, 84, 85, 86, 88, 90, 91, 92, 93, 94, 95, 96, 97, 98, 99, 101, 102, 104, 105, 106, 107, 108, 109, 110, 111, 112, 113, 114, 115, 116, 117, 118
Link node	10	5, 9, 30, 37, 38, 63, 64, 68, 71, 81

#### 5.1.3. Extreme Weather Data

Since the failure rate mentioned before in (25), here it is necessary to define the functional relationships between the ice and wind load and the failure rate connected to that. According to the literature [61], Table 4 is about the failure rate function and have been concluded as follows for the next simulation part.

**Table 3:** Failure rate function of ice and wind load.

*L_W_*	*λ_W_* (Number/h, 50 km)	*L_I_*	*λ_I_* (Number/h, 50 km)
*L_W_* ≤ 0.*_W_*	1.2 × 10^−5^	*L_I_* ≤ 0.3*dl_I_*	0
0.9*dl_W_* < *L_W_* ≤ 1.0*dl_W_*	8.0 × 10^−4^	0.3*dl_I_* < *L_I_* ≤ 0.5*dl_I_*	4.5 × 10^−3^
1.0*dl_W_* < *L_W_* ≤ 1.1*dl_W_*	0.048	0.5*dl_I_* < *L_I_* ≤ 0.9*dl_I_*	0.010
1.1*dl_W_* < *L_W_* ≤ 1.2*dl_W_*	0.060	0.9*dl_I_* < *L_I_* ≤ 1.0*dl_I_*	0.015
1.2*dl_W_* < *L_W_* ≤ 1.5*dl_W_*	0.028	1.0*dl_I_* < *L_I_* ≤ 1.1*dl_I_*	0.033
1.5*dl_W_* < *L_W_*	0.04	1.1*dl_I_* < *L_I_* ≤ 1.2*dl_I_*	0.050
/	/	1.2*dl_I_* < *L_I_* ≤ 1.5*dl_I_*	0.071
/	/	1.5*dl_I_* < *L_I_*	0.10

Now that the failure rate has been decided by the mentioned function, it is important to figure out how to conduct the extreme weather coming into the system. According to the first chapter, the topology model of the power grid is established, and the power grid is transformed into a directed weighted topology model. Figure 15 is a schematic diagram of ice and wind effects range, in which a radius is established with a unit length of their effects.

**Figure 16:**
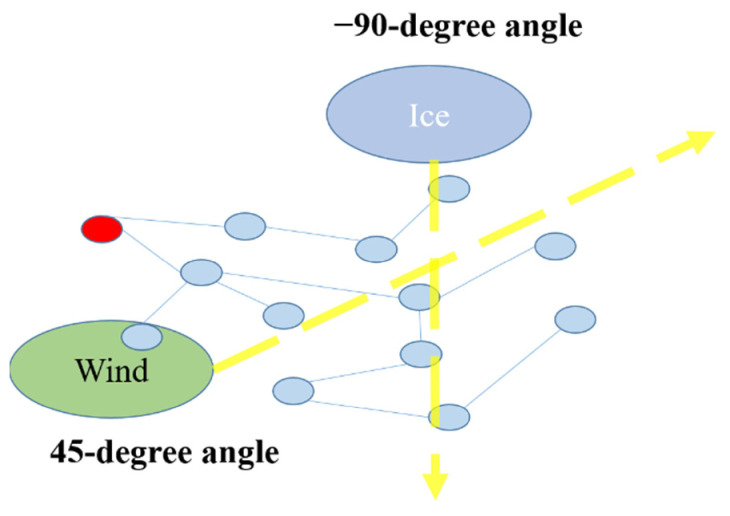
Ice and Wind started sites in the topological model.

**Figure 1:**
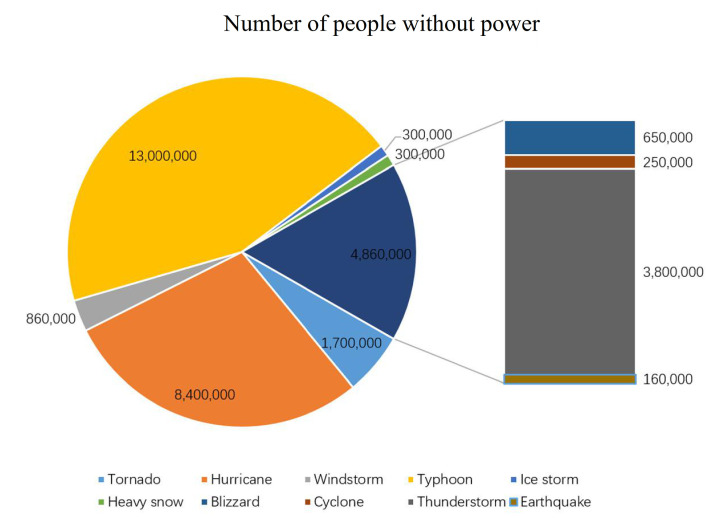
Pie chart of major power outages under extreme cases from 2011–2016.

**Figure 2:**
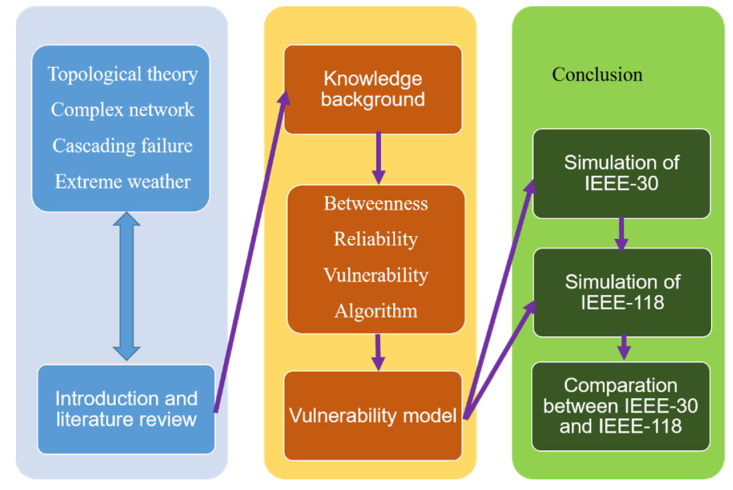
Structure flowchart of this paper.

**Figure 3:**
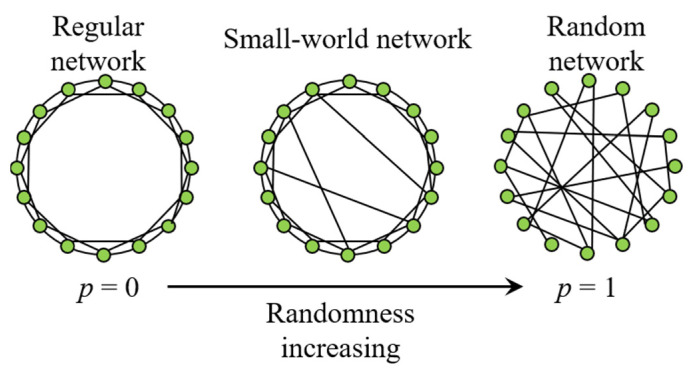
WS small world network model structure.

**Figure 4:**
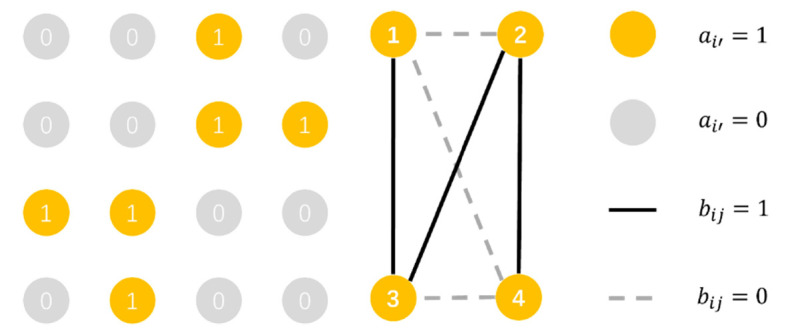
An example of undirected and unweighted graph.

**Figure 5:**
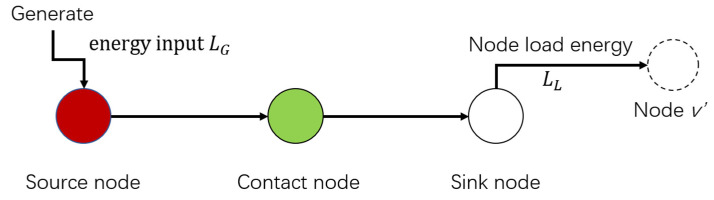
Node classification.

**Figure 6:**
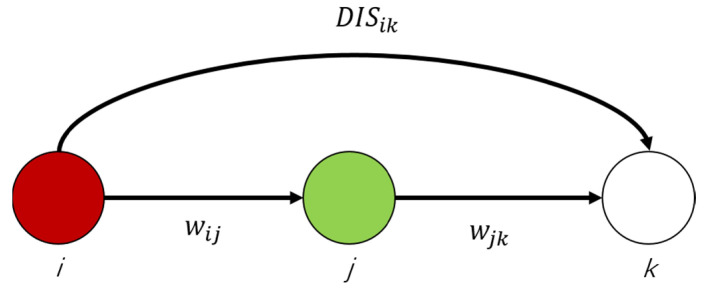
Weight calculation.

**Figure 7:**
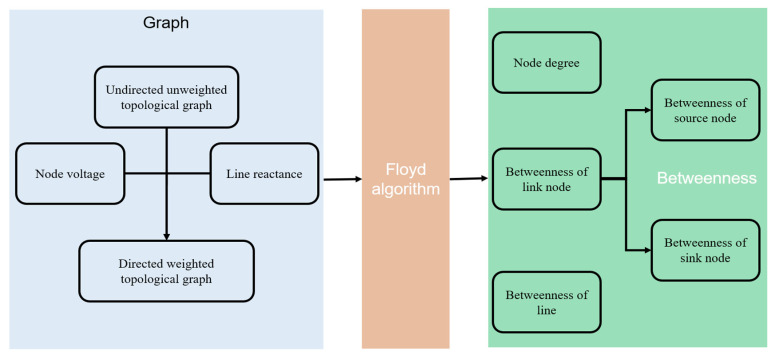
Flow chart of power grid topology modeling.

**Figure 9:**
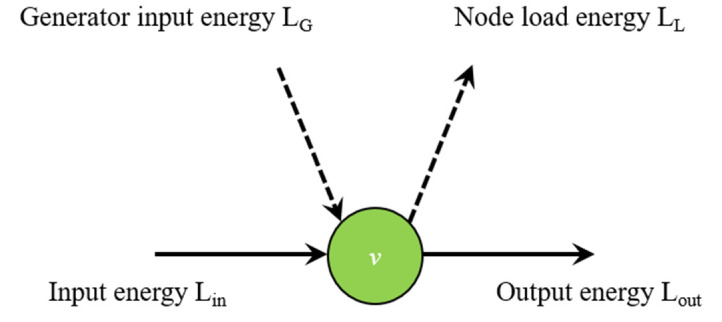
Schematic diagram of energy flowing through nodes.

**Figure 11:**
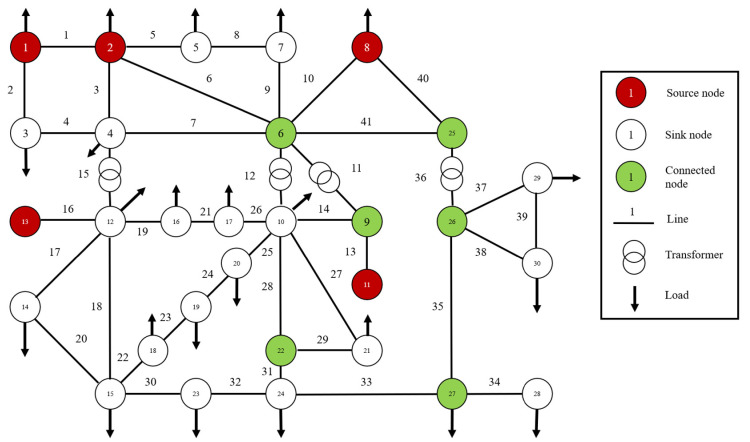
IEEE-30 bus system topology model diagram.

**Figure 15:**
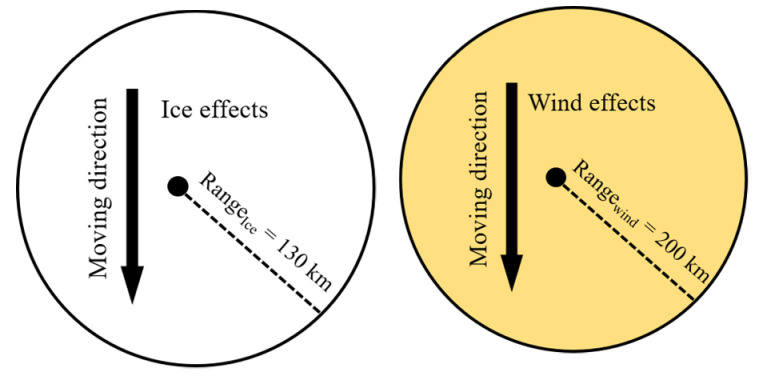
Ice effects range and Wind effects range.

To make cases much easier to understand, two kinds of extreme cases are drawn in Figure 16. They meet a simple topological network in two different angles and original start sites, which indicates similar sites in IEEE-30 and IEEE-118. In this paper, the direction of wind is 45-degrees and starts to blow in 10 km from (0,0) to the end of the map with radius equal to 200 km in the topological Figure 14. Similarly, the direction of ice is −90-degree and start to blow in 10 km from (10,18) to the end of the map with radius equal to 130 km in the topological Figure 14. By doing this, the influences caused by the two cases can be obtained easily and clearly. By utilizing (25), this paper considers the coefficients of the two proposed extreme cases at the same time and set the maximum load of ice and wind to 50 and 18.95. The wind and ice will go through the power grid from different angles and started sties to influence the lines that are on their ways. Lines within their ways will suffer from wind and ice according to its position and the distance between the line and the central of the wind or ice. After the wind passing through the lines, the load of wind and ice will gradually decrease as time goes by. In this paper, the data of extreme weather is set as Table 4 and Table 5.

**Table 4:** Load of wind and ice of highest 8 lines in IEEE-30 during this process.

Number of Lines (Wind)	Load of Wind	Number of Lines (Ice)	Load of Ice
22	23.63285903	12	65.17233427
30	22.65910081	14	65.17233427
18	22.65449605	28	65.17233422
20	21.91749813	6	65.16670399
23	18.49225909	7	65.16670399
32	12.28254813	10	65.16670399
24	6.298664663	9	65.16577223
17	1.585745099	26	65.16028429

**Table 5:** Load of wind and ice of highest 8 lines in IEEE-118 during this process.

Number of Lines (Wind)	Load of Wind	Number of Lines (Ice)	Load of Ice
110	23.61433234	46	65.16670399
114	23.53946714	49	65.16670399
108	23.53265901	54	65.1241117
109	23.53114991	108	65.00203936
115	23.52855244	109	65.00203936
112	23.48828542	114	64.96905002
111	23.35066356	110	64.96862148
113	21.02053777	115	64.95683736

### 5.2. Simulation and Analysis of Power Grid Functional Vulnerability

#### 5.2.1. Structural Vulnerability Calculation of Proposed Model without Extreme Weather

**Figure 17:**
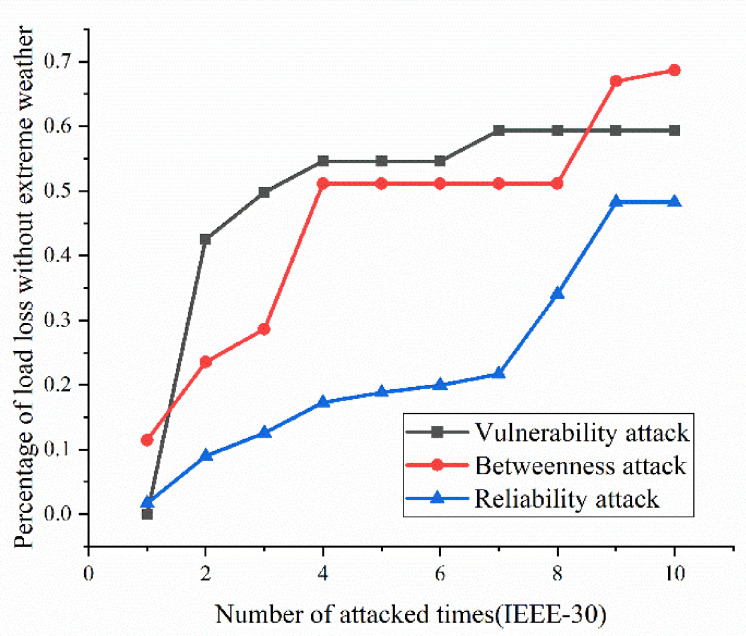
Load loss ratio of IEEE-30 bus system without extreme weather.

**Figure 18:**
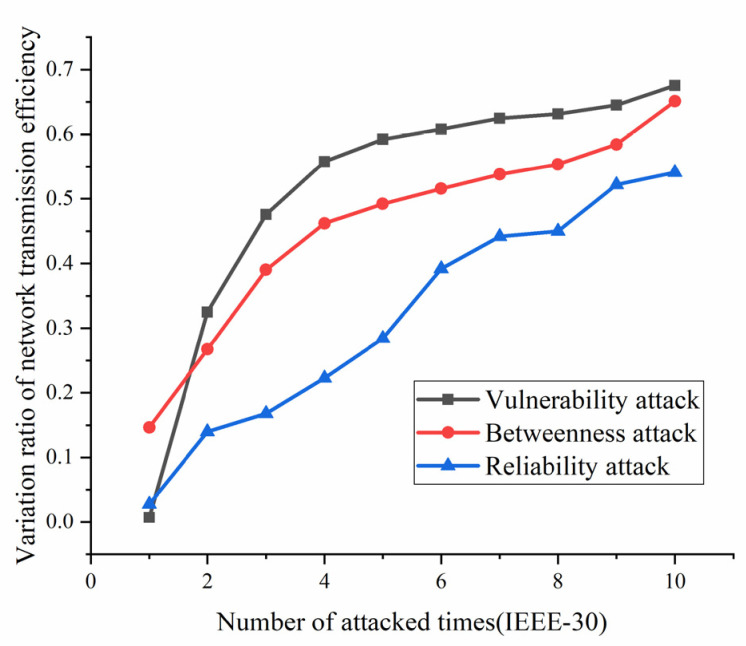
Variation ratio of network transmission efficiency of IEEE-30 bus system without extreme weather.

**Figure 19:**
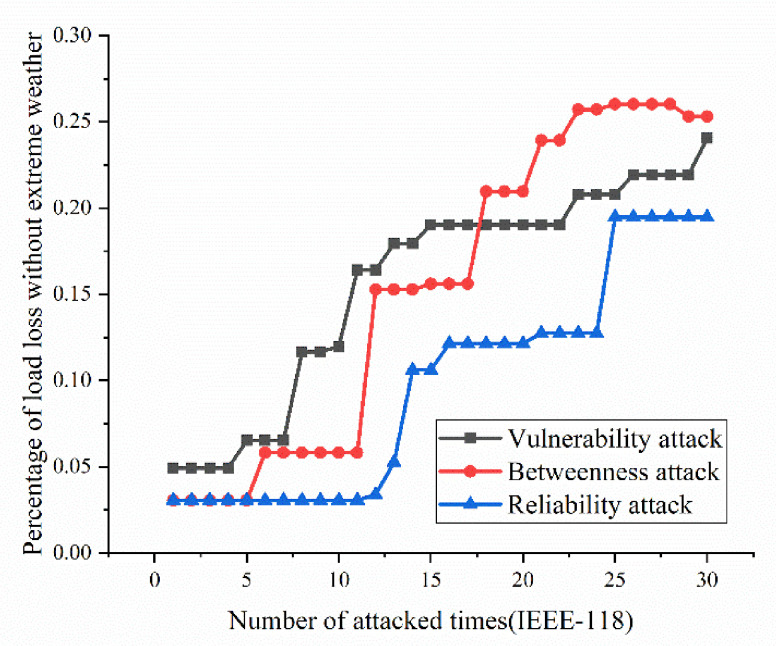
Load loss ratio of IEEE-118 bus system without extreme weather.

**Figure 20:**
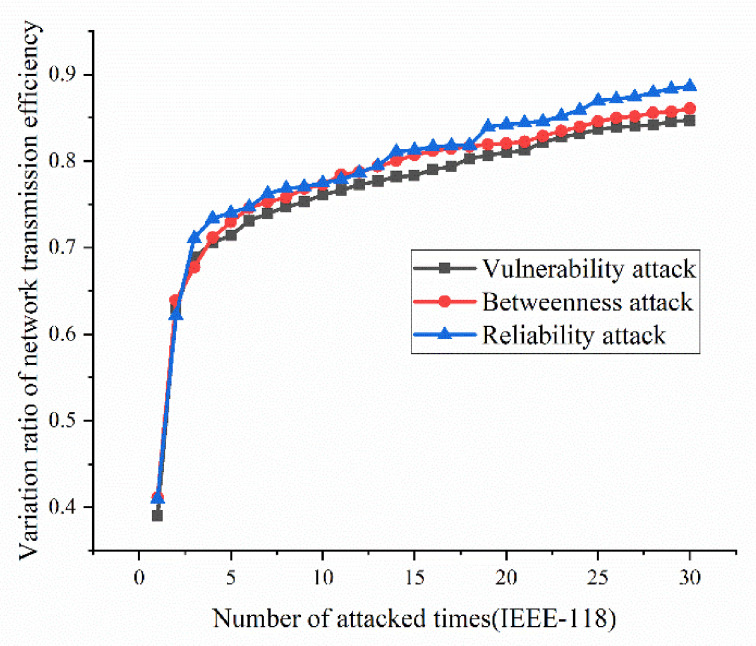
Variation ratio of network transmission efficiency of IEEE-118 bus system without extreme weather.

According to the definition and algorithm of power grid structural vulnerability in extreme cases in Chapter 3, this section mainly calculates vulnerability attack, dielectric attack, and reliability random attack results. However, at first, here the algorithm does not use the extreme cases as background to get initial version of structural vulnerability results. The results are as Figure 17, Figure 18, Figure 19 and Figure 20. Figure 17 and Figure 20 shows the power grid of 30 nodes and 118 nodes perform differently. In Figure 17, the percentage of load loss is pretty high under vulnerability and betweenness attack, while reliability attack seems to hold the weakest attack effect. However, in Figure 19, it is different. Vulnerability and betweenness attack caused load losses to shrink greatly, nearly 60%, while the load loss caused by reliability attack only decreased 30%. However, the resulting variation ratio between Figure 18 and Figure 20 is consistent. Their data indicates the variations ratio of network transmission efficiency grow fast for the first five attacks. Then it tends to slowly increase after the first 5 attacks.

#### 5.2.2. Structural Vulnerability Calculation of Proposed Model under Extreme Weather

**Figure 21:**
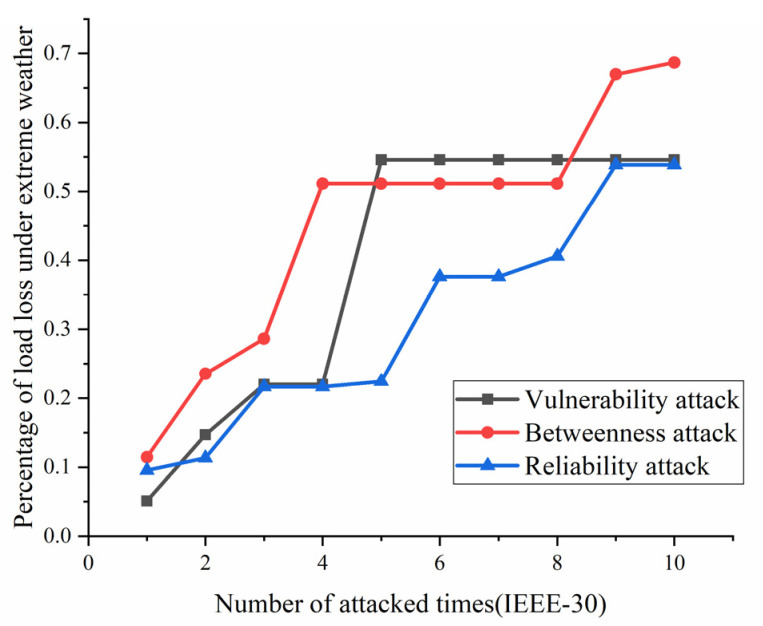
Load loss ratio of IEEE-30 bus system under extreme weather.

**Figure 22:**
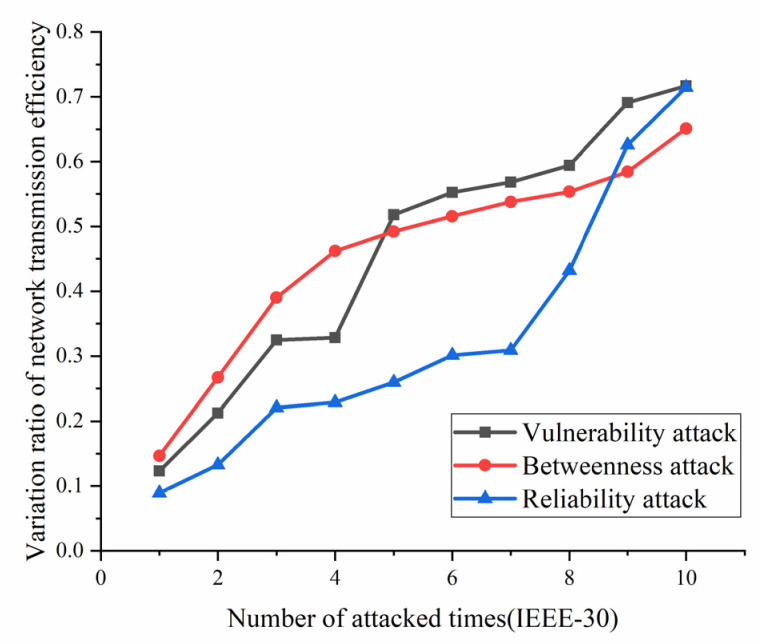
Variation ratio of network transmission efficiency of IEEE-30 bus system under extreme weather.

**Figure 23:**
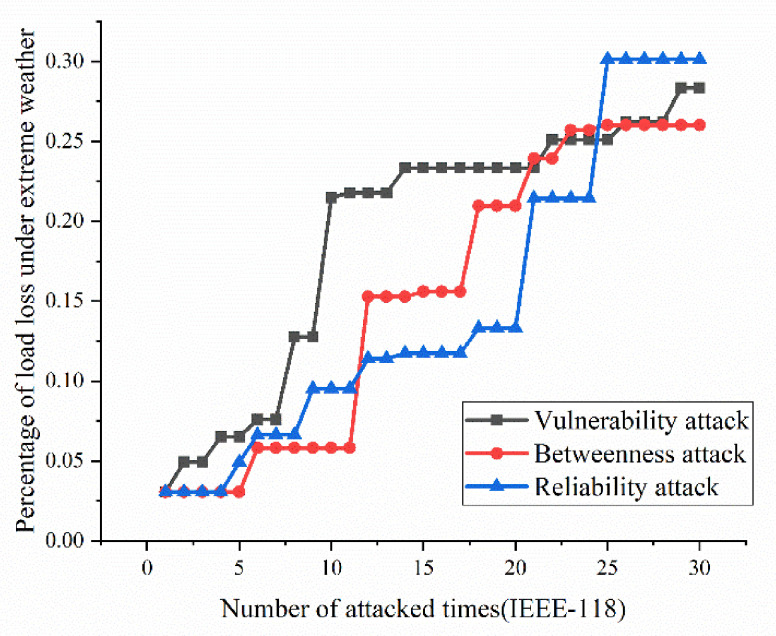
Load loss ratio of IEEE-118 bus system under extreme weather.

**Figure 24:**
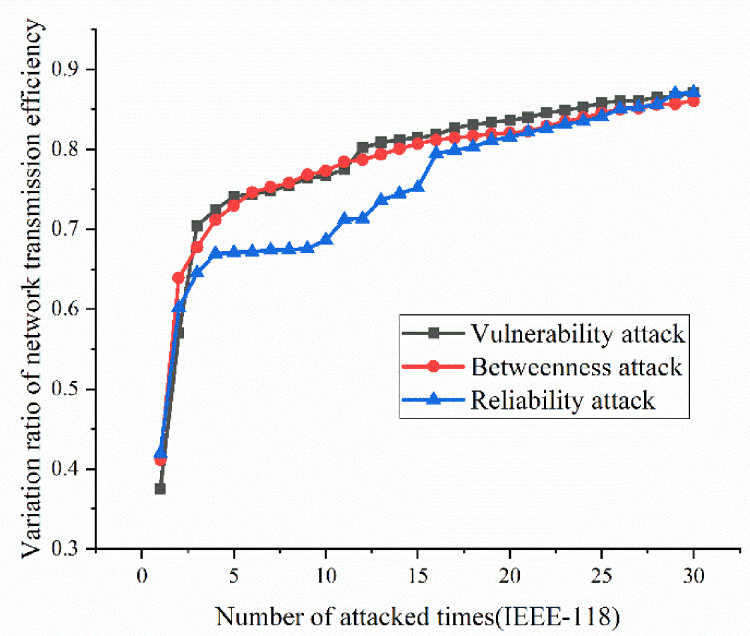
Variation ratio of network transmission efficiency of IEEE-118 bus system under extreme weather.

In this part, the extreme cases are considered by using Chapters 3 and 4. Similarly, Figure 21, Figure 22, Figure 23 and Figure 24 indicate the percentage of load loss and the percentage of network transmission efficiency decline under the three attack modes of IEEE-118 and IEEE-30 cases, respectively, under extreme weather. Compared with the Figure 17, Figure 18, Figure 19 and Figure 20, Figure 21, Figure 22, Figure 23 and Figure 24 which consider extreme weather illustrate that with extreme cases, the percentage of load loss and variation ratio of network transmission efficiency perform with higher values which indicate the damage made by extreme cases is also bigger, and the values commonly increased.

#### 5.2.3. Structural Vulnerability Result Analysis by Comparing IEEE-30 with IEEE-118

**Figure 25:**
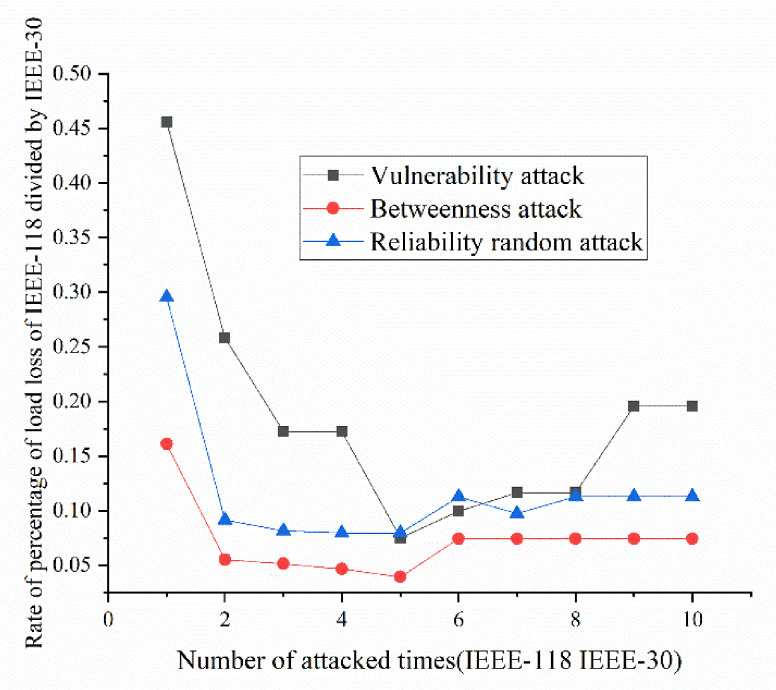
Rate of percentage of load loss under the three set attack modes.

**Figure 26:**
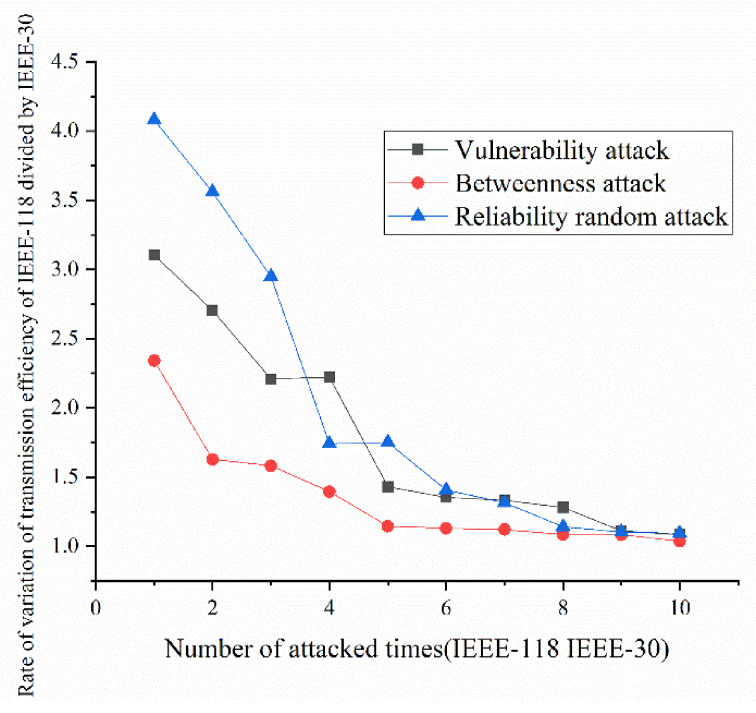
Rate of transmission efficiency under the three set attack modes.

Here, this paper proposes rates of indexes in IEEE-118 divided by indexes in IEEE-30 with the number of attacked times from 1 to 10. Thus, it is much easier to see the difference of results between the two examples. The results in Figure 25 show that the percentage of load loss of IEEE-118 is clearly much smaller than the percentage of load loss of IEEE-30, proving that bigger network IEEE-118 usually is more stable and reliable than the IEEE-30. On the contrary, in Figure 26, due to more lines and nodes need to be traverse, the variation ratio of transmission efficiency of IEEE-118 is obviously much higher than the ratio of IEEE-30, which suggests that the transmission efficiency in IEEE-118 is much more unstable. The reason why IEEE-118 is not stable in transmission efficiency is that it holds more nodes and lines. Hence, if a line or node is removed, the influenced nodes and lines are much more than the influenced nodes and lines in IEEE-30.

#### 5.2.4. Comparation with Other Literatures

**Figure 27:**
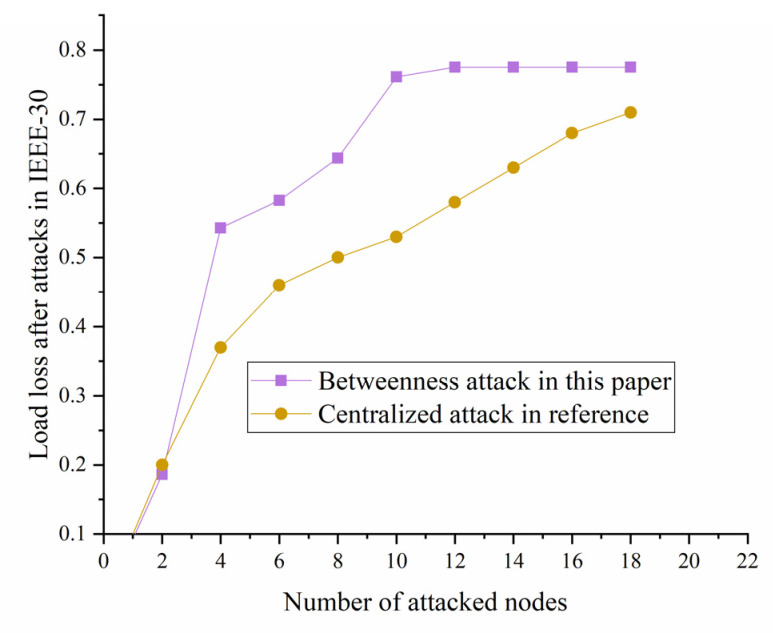
Comparation of the vulnerability with literature [62].

To evaluate the efficiency of this structural vulnerability result, it is an absolute necessity to compare this result with results from the other similar literature. In the case of utilizing the previous result, here this paper draws the diagram of IEEE-30 without extreme weather with the diagram calculated in literature [62]. Since literature [62] only uses centrality-based attacks which are utilized to remove the nodes with the highest centrality score, it is only possible to compare its result with the result of our betweenness attack with or without extreme weather as a condition. In [62], the highest centrality scores are the node with the highest degree, which is similar to betweenness in our paper, but betweenness is much more complicated for considering weighted and directed condition in Section 3. [62] considers the centralized attack modes of IEEE-30. The centralized parameter is related to betweenness and other indexes mentioned in the paper. We decided to compare the centralized attack result with our result of betweenness attack mode and get the results shown in the following diagram. It can be seen in Figure 27 that although the results from the start are different, the two load loss indexes of the two modes are getting closer and closer as the number of attacked nodes rises, proving that the result is reliable and applicable. Since the centralized attack is one improved attack mode based on betweenness in [62], there are relations for their common features. The results of IEEE-30 show close value of load loss, which proves that the betweenness in this paper and in the literature [62] are quite relative and effective. However, there are also differences for the two papers aimed at different topics, indicating that the betweenness has bigger impacts on load loss due to the extreme weather.

#### 5.2.5. Cascading Failures of the Cases Study of IEEE-30 and IEEE-118

**Figure 28:**
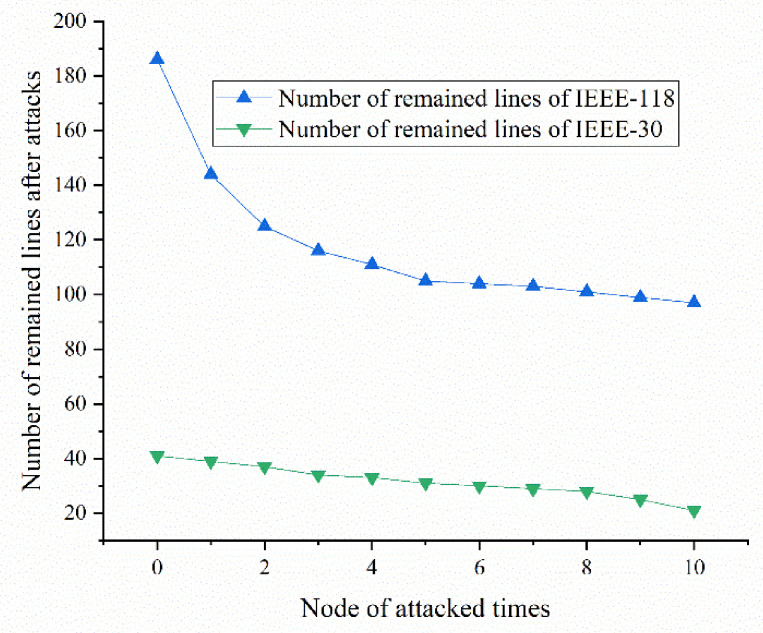
Cascading failures as attacks happening.

Cascading failures of the cases study are mainly occurring when a series of nodes are broken one after another and cause blackouts and outages. Here this part presents the failure lines with time as an x-coordinate in IEEE-30 and IEEE-118. In Figure 28, it is clear that the number of broken lines in IEEE-118 is growing faster at first. The number of broken lines of the two systems is growing at a similar speed after the first a few attacks. The number in IEEE-118 grows lower, which indicates that the IEEE-118 is much more stable after the first a few attacks. Hence, this can be used to describe the process of vulnerability for more failed lines, meaning it is much more unstable. This can be utilized as an example to understand the state of the power grid now and where it is in the power grid failure process.

## 6. Discussion

The results in IEEE-30 and IEEE-118 present high common features in trending. However, compared with IEEE-30, IEEE-118 is much stronger and more adjustable for holding more nodes and lines to manage. Furthermore, extreme cases cast greater impacts on the IEEE-30 than on IEEE-118. Extreme weather really influences the reliability much more after knowing the load loss after random reliability attacks of two systems. The main reason for this may be that in extreme weather a random attack may just occur in a fragile place, which is just influenced by extreme cases. In the future, the relationships between extreme cases and random attacks may be the next part to explore. Still, load loss and transmission efficiency really act differently. The next future topic maybe the features in topological structural classifications of parameters.

Furthermore, the extreme weather used in this paper is partly from the cited work. The extreme weather model is not very innovative. In the future, we hope to establish the extreme weather model considering the features of the power grid.

## 7. Conclusions

Power grid topology modeling based on the complex network can reflect the connection structure of power grid units and better identify the structural vulnerability of different units. The degree of node and the weighted intermediate of nodes and lines are introduced as model characteristic parameters to analyze the topology characteristics of the power grid model more effectively. Based on the structural vulnerability model of cascading failure, we propose indexes to address the vulnerability including percentage of load loss and variation ratio of network transmission efficiency. Also, we propose a structural vulnerability model considering extreme cases and analyze the relationships between power loss and power load capacity. Finally, this paper designs a relative algorithm.

The example in this paper further verifies the necessity of analyzing the vulnerability of the power grid structure under extreme circumstances, and finds out

(1) Variation ratio of network transmission efficiency grows fast at the first 5 removed nodes both in IEEE-30 and IEEE-118. Vulnerability and betweenness attack caused load losses to shrink nearly 50% from IEEE-30 to IEEE-118, while the load loss caused by reliability attack only decreases 10%. It can be seen from Figure 18, Figure 19, Figure 20, Figure 21, Figure 22, Figure 23, Figure 24, Figure 25 and Figure 26 that the power grid has good robustness against line random attack in extreme cases, and the percentage of load loss and the percentage of network transmission efficiency de-cline increase slowly in line random attack mode.
(2) The percentage of load loss and variation ratio of network transmission efficiency appear with higher values, considering extreme cases.
(3) Bigger network IEEE-118 usually performs more stably and reliably than the IEEE-30, with regard to load loss. However, the variation ratio of transmission efficiency of IEEE-118 is obviously much higher than the ratio of IEEE-30; the influenced nodes and lines are more in IEEE-118.

This paper analyzes the vulnerability of the power grid from a macroscopic perspective, ignoring the internal physical characteristics of the power grid and the dynamic characteristics of complex power grid systems. In future research, the combination of macro and micro will be considered to better study the influence of grid unit reliability on grid cascading failure.

## Figures and Tables

**Table 1:** IEEE-30 system node type.

Node Type	Node Number	Node ID
Source node	5	1, 2, 8, 11, 13
Sink node	19	3, 4, 5, 7, 10, 12, 14, 15, 16, 17, 18, 19, 20, 21, 23, 24, 28, 29, 30
Link node	6	6, 9, 22, 25, 26, 27

## Data Availability

The study used the open data.

## Nomenclature

**Symbol**	**Description**	**SI (\ represents none)**
$w_{ij}$	Weight of edge *ij*	P.U.
$DIS_{ij}$	The sum of the edge reactance included in the shortest path from node *i* to *j*.	P.U.
AL	Average distance of $DIS_{ij}$	P.U.
$BT_{v}$	Proportion of weighted shortest paths passing through a node in the network to all weighted shortest paths in the network	\
$BT_{e}$	Proportion of weighted shortest paths passing through an edge in the network to all weighted shortest paths in the network	\
$\delta_{ij}$	The number of shortest paths between node *i* and node *j*.	\
$\delta_{ij}{}_{v}$	The number of shortest paths between node *i* and node *j* containing node v	\
$\delta_{ij}{}_{e}$	the number of shortest paths between node *i* and node *j* containing edge *e*	\
$n_{L}$	The number of sink nodes	\
$n_{G}$	The number of source nodes	\
$k_{i}$	The degree of node *i*	
AVED	The average degree of all node degree	\
$L_{j}$	the load of *jth* node	MV
$\eta_{L}$	The ratio of the load of all normal operation sink nodes in the current power grid to the load of all nodes in the power grid in the initial state	\
E	the average of the reciprocal distance between the network nodes of all node pairs in the power grid	\
$\eta_{E}$	The ratio between the current network transmission efficiency *E* and the initial network transmission efficiency $E_{0}$	\
$L_{in}$	Input energy	MV
$L_{out}$	Output energy	MV
$S_{Rated}{}_{i}$	Rated load capacity	MV
$S_{Limit}{}_{i}$	Limited rated load capacity	MV
$a_{0}$	Rated load capacity coefficient	\
$a_{1}$	Limited rated load capacity coefficient	\
$wire_{x_{i}}$	Position of x-coordinate of line *i* in topological graph	KM
$wire_{yi}$	Position of y-coordinate of line *i* in topological graph	KM
$EW_{x}$	Position of x-coordinate of the extreme case in topological graph	KM
$EW_{y}$	Position of y-coordinate of the extreme case in topological graph	KM
r	Radius of impact range	KM
∂	Coefficient	\
$\lambda_{I}$	Failure rate of extreme weather *I*	\
$a_{I}$	Coefficient of weather *I*	\
